# Lung Cancer Classification in Histopathology Images Using Multiresolution Efficient Nets

**DOI:** 10.1155/2023/7282944

**Published:** 2023-10-16

**Authors:** Sunila Anjum, Imran Ahmed, Muhammad Asif, Hanan Aljuaid, Fahad Alturise, Yazeed Yasin Ghadi, Rashad Elhabob

**Affiliations:** ^1^Center of Excellence in Information Technology, Institute of Management Sciences, Hayatabad, Peshawar 25000, Pakistan; ^2^School of Computing and Information Science, Anglia Ruskin University, Cambridge, UK; ^3^Department of Computer Science, National Textile University, Faisalabad, Pakistan; ^4^Computer Sciences Department, College of Computer and Information Sciences, Princess Nourah bint Abdulrahman University (PNU), P.O. Box 84428, Riyadh 11671, Saudi Arabia; ^5^Department of Computer, College of Science and Arts in Ar Rass, Qassim University, Ar Rass, Qassim, Saudi Arabia; ^6^Department of Software Engineering/Computer Science, Al Ain University, Al Ain, UAE; ^7^College of Computer Science and Information Technology, Karary University, Omdurman, Sudan

## Abstract

Histopathological images are very effective for investigating the status of various biological structures and diagnosing diseases like cancer. In addition, digital histopathology increases diagnosis precision and provides better image quality and more detail for the pathologist with multiple viewing options and team annotations. As a result of the benefits above, faster treatment is available, increasing therapy success rates and patient recovery and survival chances. However, the present manual examination of these images is tedious and time-consuming for pathologists. Therefore, reliable automated techniques are needed to effectively classify normal and malignant cancer images. This paper applied a deep learning approach, namely, EfficientNet and its variants from B0 to B7. We used different image resolutions for each model, from 224 × 224 pixels to 600 × 600 pixels. We also applied transfer learning and parameter tuning techniques to improve the results and overcome the overfitting problem. We collected the dataset from the Lung and Colon Cancer Histopathological Image LC25000 image dataset. The dataset acquisition consists of 25,000 histopathology images of five classes (lung adenocarcinoma, lung squamous cell carcinoma, benign lung tissue, colon adenocarcinoma, and colon benign tissue). Then, we performed preprocessing on the dataset to remove the noisy images and bring them into a standard format. The model's performance was evaluated in terms of classification accuracy and loss. We have achieved good accuracy results for all variants; however, the results of EfficientNetB2 stand excellent, with an accuracy of 97% for 260 × 260 pixels resolution images.

## 1. Introduction

Malignant growth has been described as a combination of related infections, including unusual cell development that continuously spreads into encompassing tissues. Different cancer types can occur in human bodies as per worldwide disease statistics [1]; lung and colon malignancies are among the most common, with lung cancer first in men and third in ladies. Colon malignancy is positioned third in men and second in ladies. The leading causes of lung cancer are tobacco and smoking, whereas the causes of colon cancer are older age, smoking, and regular use of red meat. Lung cancer subtypes are lung adenocarcinoma, lung squamous cell carcinoma, and colon adenocarcinoma [2], identified by histopathology, which studies tissues using a microscope. A histopathology report is called a biopsy report, in which the doctor identifies subtypes of cancer and their stage. The characteristics and treatments of different pathologic subtypes of cancer are dissimilar. Nevertheless, the right and on-time prognosis can execute a compelling treatment design and draw outpatient endurance.

Computer-aided diagnosis (CAD) systems can be a helpful tool for avoiding misclassification [3]. The CAD gives a modernized yield as a “second assessment” to help a pathologist's findings and helps clinical technologists and pathologists to assess malignancies more precisely. Artificial intelligence approaches have improved the precision and computerization of histopathologic slide examination. Convolutional neural networks (CNNs) are presently the best method to assemble dynamic work processes in computerized pathology [3]. When a given CNN model is adequately trained on labelled image information, it takes complex histological features from pictures through deconvolution of the picture content, which has many features and afterwards perceives these features in inconspicuous pictures. Any basic CNN model is based upon convolutional filters, pooling, and fully connected layers. Different hidden layers of CNN give various picture details level information to detect complex features.

The major goal of this work is to categorize lung and colon cancer biopsy images into five classes with subtypes of lung adenocarcinoma, squamous cell carcinoma, normal tissues, colon adenocarcinoma, and colon normal tissues using the EfficientNet model to observe the effect of the CNN model when image resolution is increased. For classifying any CNN model, a large dataset is usually required. Our chosen dataset consists of twenty-five thousand images with five classes, but it is not enough. CNN architecture faces the problem of overfitting due to the small size of the dataset. Pretrained models with fine turning can be used to prevent overfitting issues and computing power. The whole dataset is divided into three phases, training, validation, and testing, for the experiment. In this work, CNN architecture with pretrained EfficientNet variants EfficientNetB0–B7 has been used to classify into five classes for lung and colon cancer histology images.

### 1.1. Objectives and Contribution of the Proposed Work

The major contribution of this research work is as follows:

1. To classify histology images into three classes of lung cancer and two classes of colon cancer with high accuracy
2. To avoid overfitting problems and train the model with limited available resources, pretrained models with fine-tuning and transfer learning techniques are used to classify images into five categories correctly
3. To find the model's effectiveness by increasing the resolution correspondingly, different variants of EfficientNet from B0 to B7 are trained, with each model having a different image resolution (from 224 × 224 pixels increased to 600 × 600 pixels).
4. To make a comparison among the proposed model and variants of EfficientNet

The related work is briefly discussed in Section 2.  Section 3 discusses the Materials and Methods. Results and Discussion are elaborated in Sections 4 and 5, followed by conclusion and future directions in Section 6.

## 2. Related Work

Various works have been found in the literature to detect, segment automatically, and classify cancerous and noncancerous from histopathology images using machine learning and deep learning techniques. Deep learning is the most recently used technique for classification tasks due to its accuracy and automatic selection of the best features. Barker et al. [4] proposed a method for cerebrum tumours in entire slide computerized pathology pictures. Ojansivu et al. [5] researched the grouping of bosom disease from histopathological images. In [6], the authors investigated different resolutions EfficientNets for sore skin grouping, joined with broad data increase, and balancing loss and considered multiresolution a significantly important parameter for the model.

In [7], the authors proposed and assessed a convolutional neural network to arrange interstitial lung infection disease patterns. The proposed network comprises five convolutional layers with two parts and Leaky ReLU, trailed by average pooling with a size equivalent to the size of the last component guides and three deep layers. In [8], lung cancer pathology subtypes are classified in CT scan images using a deep residual neural network with transfer learning techniques which achieved 85% of accuracy. In [9–12], researchers applied different models to classify lung cancer types or lung cancer from noncancer images with various deep learning models and tried to increase the model's accuracy using other datasets. Iizuka et al. [13] used the inception v3 model and the recurrent neural network to classify stomach and colon biopsy histopathology from whole slide images. The model was trained to classify adenocarcinoma, adenoma, and nonneoplastic. In addition, the authors added regularization methods and different augmentation techniques to make the algorithm more robust. Rathore et al. [14] used an SVM classifier to examine colon cancer of histopathology images in normal and malignant tissue.

The proposed approach [15] was tried on a histopathological dataset for colorectal malignancy order in light of seven sorts of CNNs. Scaling up CNN models is broadly used to improve accuracy [16]. The most basic route is the scale-up CNN model by depth [17]; these networks are simpler to advance and can acquire precision from an impressively expanded depth or scale model through width [18]. Another more uncommon, however, progressively mainstream technique is scaling up models by increasing the size of images [19]. The EfficientNet model [16] addresses all three scaling methods named compound scaling. However, the model needs more layers to build the open field and channels to catch all the fine-grained designs on the larger picture for the larger input image size. Moreover, increasing image resolution [20] compromises the largest conceivable batch size for CNN training. After reviewing the literature, this paper presented a method to classify the colon and lung histopathology images using all variants of the EfficientNet model (from B0 to B1), increasing the image resolution up to 600 × 600 pixels.

## 3. Materials and Methods

This section explains our proposed methodology, experiment, and dataset used to classify lung and colon histopathology images using EfficientNet Model variants from B0 to B7.

### 3.1. Images Dataset Acquisition

**Figure 1:**
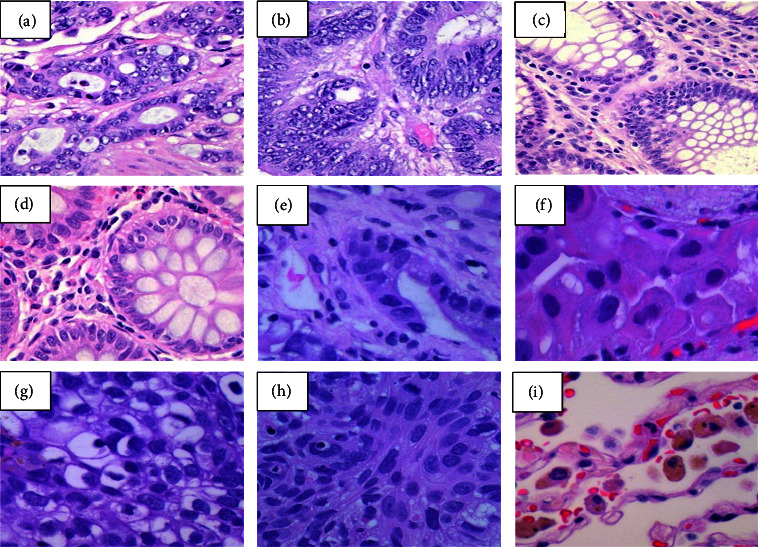
Image samples from LC25000 dataset image. (a, b) Colon adenocarcinoma. (c, d) Colon benign tissue. (e, f) Lung adenocarcinoma. (g, h) Lung squamous cell carcinomas. (i) Benign lung tissue.

The dataset used in this research is taken from the Lung and Colon Cancer Histopathological Image LC25000 image dataset [21], which consists of 25,000 images with two subfolders: colon cancer folders containing 10,000 images and lung cancer folders with 15,000 images. All images are in JPEG format and are 768 × 768 pixels in size. The lung cancer folder contains three subfolders with two lung cancer types and benign lung tissue images. From histopathology images, cancerous and noncancerous can be identified as follows:

1. Malignant tissue: It can be identified as dark in colour and abnormal nuclei tissue growth compared to its normal tissue image, as shown in Figure 1.
2. Benign tissue: This region has normal growth of tissues and is light in colour.

Three functions are applied to augment the images, as explained in [21]; the first function is rotated randomly between 25% on the right and 25% on the left. The second function adds random noise to the images, and the third function is horizontal flips, which flips the image array of pixels. Using these augmentation functions, the images in the dataset are expanded for lung cancer images up to 15,000. So, in this research, the five classes, lung adenocarcinoma, lung squamous cell carcinomas, benign lung tissue, colon adenocarcinoma, and colon benign tissue, are considered to be classified. Each class contains 5,000 images. The same for colon images; make 10,000 images, each of the two categories having 5,000 images.

### 3.2. Preprocessing

**Figure 2:**
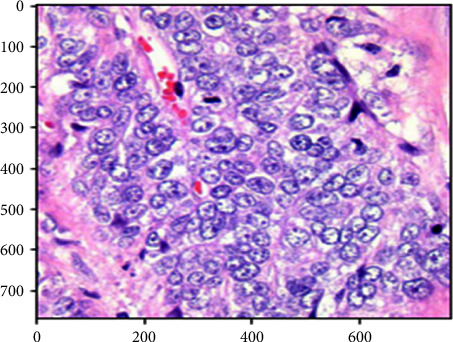
Index representation of the dataset image sample before preprocessing to the model's desired size.

In the preprocessing phase, we have to ensure that all images are equal in size for the best result of the CNN model. In the first step, all images are in 768 × 768 pixels, as shown in Figure 2. We adjusted the image resolution size as required for each EfficientNet model variant according to Table 1. As for EfficientNetB0, convert all images into equal sizes of 224 × 224 pixels for all training, validation, and testing stages. To avoid the imbalanced problem [22], the classification of classes is not balanced; that is, one class has more images than all other classes, so we must have an equal number of images in each class. That is why each class has an equivalent of 5,000 images in our dataset. A straightforward way to deal with surviving class irregularities in model learning is to resample the training data (a premeasure).

The whole dataset is divided for the CNN model's training, validation, and testing phases in the second step. To avoid the overfitting problem (which means not having enough data in the training phase that the model does not correctly predict the classes) and for the best accuracy results, more images are kept in a training folder of 25,000 images, 15,000 images belonging to 5 categories (colon adenocarcinoma, benign colon tissue, lung adenocarcinoma, lung benign, and lung squamous cell carcinoma). In the validation folder, we allocate 5000 images belonging to 5 classes and assign 5000 images belonging to 5 classes for testing. In the third and last step of the preprocessing phase, we make sure that all images in subfolders are correctly labelled; all images in the colon adenocarcinoma folder are labelled as “colonca1” 1 represents the number of an image, and in the same way, all images in a colon benign tissue folder are marked as “colonn1”. Also, all images in three classes of lung folders are differently labelled (lungaca1, lungn1, and lungscc1); this helps our model in the ImageDataGenerator phase to train and generate labels for the training phase.

### 3.3. Transfer Learning

**Figure 3:**
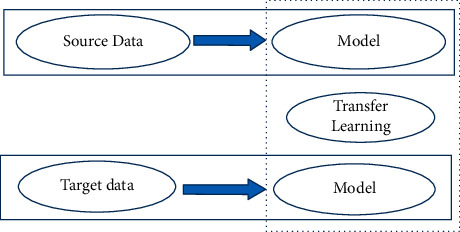
Transfer learning schematic diagram.

Transfer learning is a mainstream approach in computer vision-related problems; hence, we combined the pretrained models with the newly trained layer of CNN architecture. Here, we are using a deep learning pretrained EfficientNet model, where the last layer of the model performs as input data to another classifier. In addition, we utilized the ImageNet dataset for transfer learning techniques for classification, which helped us achieve better performance accuracy and saved training time.  Figure 3 shows the schematic diagram for transfer learning. It can be seen that the previous model that was trained on source data is combined with the newly trained target data model with the help of transfer learning.

### 3.4. Proposed CNN Method

**Figure 4:**
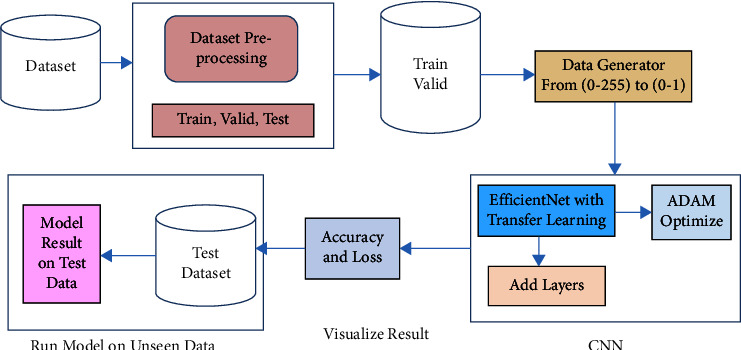
Block diagram of the proposed work.

This section explains our proposed work for classifying lung and colon cancer into five classes (lung benign, lung adenocarcinomas, lung squamous cell carcinomas, colon adenocarcinomas, and colon benign), as shown in Figure 4. Transfer learning on the ImageNet dataset and the fine-tuning method means adjusting each model's last layers to achieve good accuracy and performance. The whole dataset is divided into three portions, where 80% of the data is used for training, 10% for validation, and 10% for testing. EfficientNet with different variants from B0 to B7 is used. Similar size images are used for each model, as mentioned in Table 1, so we first resize it into 224 × 224 pixels for EfficientNet B0 and the same for other models. After preprocessing and training, the model results are evaluated.

### 3.5. EfficientNet and Variants

**Figure 5:**
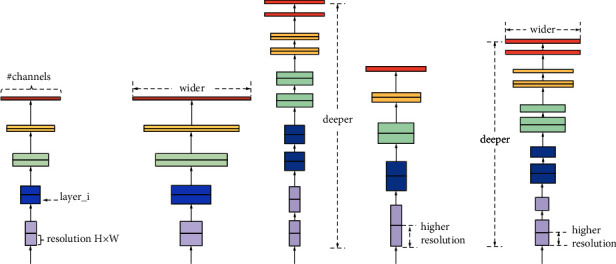
The basic idea of EfficientNet [16] is to carefully balance scale, the network width depth, and resolution if resources are available (https://ai.googleblog.com/2019/05/efficientnet-improving-accuracy-and.html).

The EfficientNet models [16] depend on straightforward and compelling compound scaling strategies. EfficientNets are a group of neural organization structures delivered by Google in 2019, planned by an enhancement methodology that amplifies the precision for a given computational expense. EfficientNets are suggested for characterization errands. They beat numerous organizations (such as DenseNet, Inception, and ResNet) on the ImageNet benchmark while running quicker. This strategy empowers the scaling up of a benchmark ConvNet to any objective asset imperative while maintaining model proficiency, which is utilized for moving learning datasets. As a rule, EfficientNet models accomplish higher precision and better productivity over existing CNNs, for example, AlexNet, ImageNet, GoogleNet, and MobileNetV2. Specifically, EfficientNet explores the focal inquiry: is there a standard technique to scale up ConvNets to accomplish better exactness and productivity? In this model, observational investigation, as shown in Figure 5, is essential to adjust all measurements of organization width (more filters in the layer), depth (more layers), and resolution (more H ∗ W); such equilibrium can be accomplished by essentially scaling each of them with steady proportion. This model is a basic yet powerful compound scaling strategy in light of this perception. Unlike all traditional models that scale any of these elements, our approach consistently scales network width, depth, and resolution with a bunch of fixed scaling coefficients.

A ConvNet Layer *I* can be characterized as a capacity:

(1) $$\begin{array}{c} Y_{i}=F_{i}\left(X_{i}\right), \end{array}$$

where *F*_*i*_ is the operator, *Y*_*i*_ is the yield tensor, and *X*_*i*_ is the input tensor with a tensor shape (*H*_*i*_, *W*_*i*_, *C*_*i*_), where *H*_*i*_ and *W*_*i*_ are the spatial measurements and Ci is the channel measurement. A rundown of created layers can address a ConvNet N:

(2) $$\begin{array}{c} N=F_{k}ʘ\ldots ʘF_{1}ʘF_{1}\left(X_{1}\right)=ʘJ_{j}=1\ldots kF_{j}\left(X1\right). \end{array}$$

The network scales the existing baseline ResNet model as described in the following equation:

(3) $$\begin{array}{c} N={\;}_{i=1\ldots s}^{ʘ}f_{i}^{L_{i}}\left(X_{\left(H_{i}W_{i}G_{i}\right)}\right), \end{array}$$

where *f*_*i*_^*L*_*i*_^ denotes layer *f*_*i*_ is repeated *L*_*i*_ times in stage *i*and (*H*_*i*_*W*_*i*_*G*_*i*_) indicates the shape of the input tensor *X* of a layer. Different variants of EfficientNet are available from B0 to B7. Each variant is scaled up to increase all three elements (depth, width, and resolution) from the previous one to see the model result in terms of accuracy and computational cost. Each number addresses variants with more parameters and higher accuracy, and the processing power generally increases for each addition. Attempt EfficientNetB0 first since its exactness is comparable to different networks while being absurdly quick to run and train. If you need to improve your outcomes, take a stab at utilizing greater and greater sizes of the EfficientNet design (B1⟶, B2⟶, B3⟶, and so on) until you hit the most elevated exactness for your information. Each model was pretrained on the ImageNet dataset Top1 and Top5 accuracy in Table 2.

## 4. Experiments

In this paper, we adopt the method to explicitly preserve the previous convolution and pooling layers, in which the model parameters of the record stacked on the dataset of ImageNet are pretrained to introduce the new network. Furthermore, the pretrained model carried out a new job by using parameters, fully associated layers, and Softmax activation function in combination with turning the last layers of the model using fine-tuning methods for each model to improve the accuracy and decrease loss. Thus, the network construction could adjust to the new characterization task in this manner, speeding and simplifying the learning effectiveness of the model and upgrading the inference capacity.

We have utilized all EfficientNet variants from B0 to B1 models for the transfer learning measure and added a batch normalization layer. Batch normalization incredibly speeds up the training of deep networks and builds the stability of the neural model [23] to limit overfitting by reducing the all-out number of parameters. Moreover, after flattening the layer, two dense inward layers with RELU activation function having 512 neurons to activate (change activates several neurons for each model) and dropout layers have been added. A 30% dropout rate has been picked randomly to overcome overfitting. At last, one dense output layer contains five output units for multiclass order to classify five classes of our dataset. Softmax implementation has been added to the proposed automatic finding framework.

We give input shape 224 × 224 size to the EfficientNetB0 model in which the dataset is divided into three parts. The training set consists of 20,000 images for five classes; validation consists of 2500; and the testing set also has 2500 images and then initializes weight as ImageNet. We keep the batch size minimum as the model takes minimum training time. Therefore, the batch size is set to 30 for the training and validation path. We change the resolution in every model as variants improve from the previous model and change the resolution size to check the model's performance efficiency.

### 4.1. Parameter Adjustment

Parameter adjustment incredibly affects the exhibition of the model since they straightforwardly administer the model's training. Also, fine-tuning can avoid overfitting and the structure of a summed-up model. Therefore, in our study, for the correct classification of lung and colon cancer histology images into five classes, a more effective CNN pretrained model, EfficientNet, is chosen. Our proposed method for this model consists of two parts.

1. First, we download and import EfficientNet and then specify the model variants.
2. Fine-tuned, fully connected layers.

In the first step, variants of EfficientNet (EfficientNetB0, EfficientNetB1, EfficientNetB2, EfficientNetB3, EfficientNetB4, EfficientNetB5, EfficientNetB6, and EfficientNetB7) are added with setting weight to ImageNet. In the fully connected layer (FCL), “Softmax” is used as an activation function. The reason for using Softmax is that the classification is categorical.

One major problem we faced during training the model was that training accuracy was not good and loss also increased. To overcome this, the last layer's parameters are adjusted to improve accuracy. The parameters are turned accordingly; when the model gives us more loss than accuracy in some variants, the dropout function is increased, in this way, the overfitting problem is reduced, and loss is decreased.

### 4.2. Implementation Details

All models are trained for 100 epochs using Adam. Categorical cross-entropy is used for the loss function in multiclass classification, one class in many possible categories. Batch size and learning rate are received depending on every network's GPU memory necessities. All the software and libraries utilized in the proposed work are open source. The perusers should use Google Colab Notebook to replicate the outcomes using the GPU run time. This product can be used without costs, since Google gives it to explore exercises utilizing a Tesla K80 GPU of 12 GB. The EfficientNet models are pre-prepared, scaled CNN models that can be used for transfer learning in picture characterization issues. The model was created by Google AI in May 2019 and is accessible through GitHub vaults. This work has been performed using Python. The neural network library “Keras” develops, compiles, and assesses the proposed methodology. The Python programming language rendition 2.7 (counting libraries, for example, numpy, cv2, pandas, and matplotlib) was utilized for all parts of this undertaking. The model was trained and tested on the framework of the window. The performance can also depend on how many medical images are loaded for training and validation.

## 5. Results and Discussion

To assess the model's performance, as per the attributes of the network model, the accuracy rate and loss rate are utilized as the evaluation measures. The normal exactness of the model is characterized as follows [24]:

(4) $$\begin{array}{c} \text{Accuracy}=\frac{R_{A}}{R}\;*100. \end{array}$$

In (4), *R* represents the total number of images in the training and validation phase, and *R*_*A*_ represents correctly classified images. The model runs for 100 epochs, with seven iterations for training and three for validation in each epoch to improve the model performance. As in the start of model training, the validation loss and training loss value is high, 40 or 50%, and then gradually decreases in each epoch. The result of every model is described in Table 3. Training results for EfficinetNetB0 are 95% accuracy and 0.37 loss, having 224 × 224 image resolution; EfficinetNetB1 is 96% accuracy and 0.11 loss for 240 × 240 image size; EfficinetNetB2 is 97% accuracy and 0.07 loss for having 260 × 260 resolution; for B3, B4, B5, B6, and B7 accuracies are 95, 94, 93, and 95% and losses are 0.18, 0.33, 0.22, and 3.05, increasing the image resolution. So, from this result, we can say that the B2 model gives us good accuracy with less loss, and image resolution is also not very minimal. B6 and B7 also have good results in terms of their image resolution size and effectiveness in being run with a Colab GPU in less time than other studies that take days to train on this resolution size and have some good physical hardware attached to the GPU.

**Figure 6:**
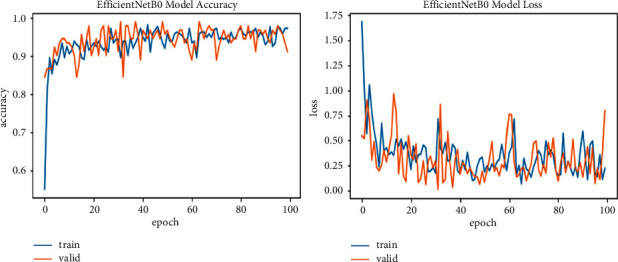
EfficientNetB0 training and validation plot of accuracy and loss.

**Figure 7:**
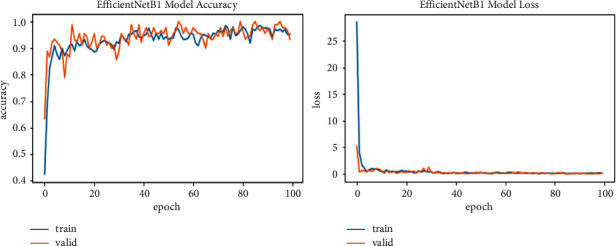
EfficientNetB1 training and validation plot of accuracy and loss.

**Figure 8:**
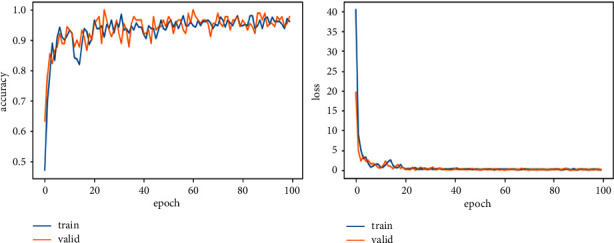
EfficientNetB2 model training and validation plot of accuracy and loss.

**Figure 9:**
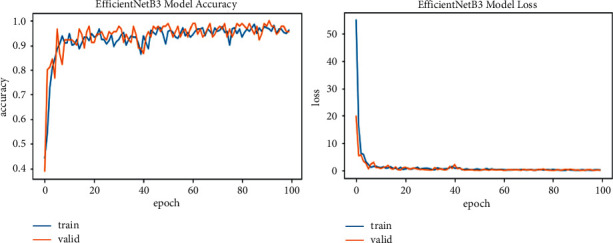
EfficientNetB3 model training and validation plot of accuracy and loss.

**Figure 10:**
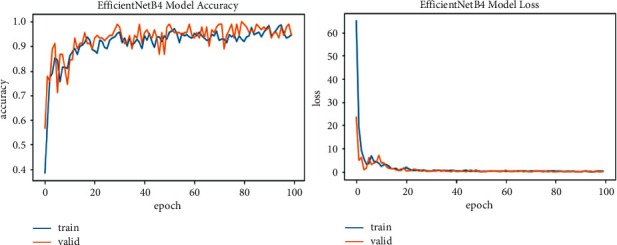
EfficientNetB4 model training and validation plot of accuracy and loss.

**Figure 11:**
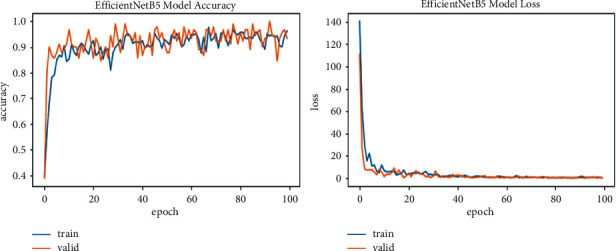
EfficientNetB5 model training and validation plot of accuracy and loss.

**Figure 12:**
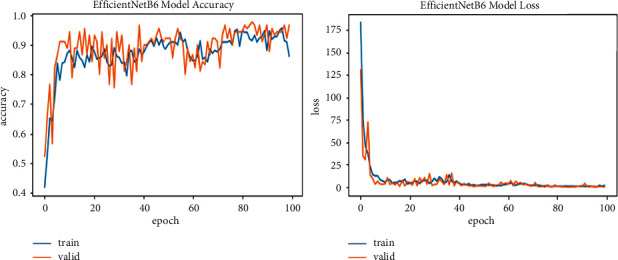
EfficientNetB6 model training and validation plot of accuracy and loss.

**Figure 13:**
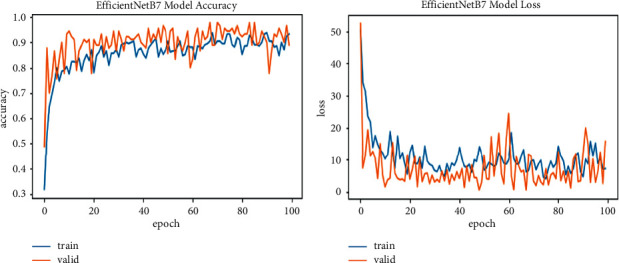
EfficientNetB7 model training and validation plot of accuracy and loss.

We explore all variants of efficient models for different image resolutions for the classification of lung and colon histopathology images into five classes (colon adenocarcinoma, benign colon tissue, lung adenocarcinoma, lung benign, and lung squamous cell carcinomas). In previous studies, these model variants were not explored for histopathology image classification, and we consider that input resolution is an important parameter that was not considered in previous studies. For a long time, small image sizes have been considered for convolutional neuron network models to increase model performance effectively. A similar study [6] increased input resolution to 528 × 528 pixels, and we increased it to 600 × 600 pixels. When the image resolution increases, more features are extracted with more fine details than fewer resolution images (as the visualization graph from B0 to B7 is shown in Figures 6–13). Our results predict that by increasing the input size, the performance does not decrease. Still, the loss is a little bit high for low-resolution sizes. Validation loss rates for B7 drop from 52.51%, 5.11%, and 2.55% after 1, 30, and 99 epochs. But this loss can also be overcome with the availability of increased memory and computation power for running more epochs.

The other measure we consider for evaluating these models' performance is the time taken to train a model for 100 epochs. The time taken by each model depends upon the Internet speed and the availability of the GPU in Colab. Sometimes the Colab is not assigning GPU to our notebook or cannot connect with the host, so we run it without GPU, and it takes many hours to train the model. Convolutional neural organizations (CNN) are neural organizations that are especially fit for picture classification. It has been, for instance, effectively utilized for picture classification orders [25–27]. A typical CNN design contains convolutional, pooling, and completely associated layers. Moderately novel procedures, for example, batch standardization [23], dropout [28], and alternate way associations [17], can, moreover, be utilized to build classification precision. In light of the outcomes, we see that the EfficientNet models, all variants with transfer learning methods, yield better results than the previous model with a simple minimum image size and many other classifier models in calculating malignancy expectation. However, on-time and accurate prognoses are challenging because of the malignancy's intricacy and high mortality. Accordingly, improving the forecast exactness by applying computer-aided diagnosis methods is very helpful for malignancy treatment.

### 5.1. Comparison with State-of-the-Artwork

In Table 4, EfficientNet variants B2 are compared with the published state-of-the-art methods used for lung and colon classification of histopathology images to prove the superiority of our approach. The EfficientNet model gives good accuracy for maximum resolution and has a minimum number of parameters compared to other models. In the literature, no one attempts to classify the lung and colon cancer images accurately [29]. In [27], the authors classified COVID-19 from X-ray pictures using EfficientNetB4, which can categorize binary and multiclass data. The deep convolutional neural network [3, 31, 32] has 60 million parameters to classify lung cancer subtypes (adenocarcinoma, squamous cell carcinoma, and small cell carcinoma), and we classify five classes. Reference [3] built up a mechanized characterization plot for cellular breakdowns in the lungs using microscopic images to utilize a deep convolutional neural organization (DCNN), an effective deep learning method. The DCNN utilized for grouping comprises three convolutional layers, three pooling layers, and two completely associated layers. Therefore, from these studies, we can say that our model result efficiently classifies the cancer images.

## 6. Conclusion and Future Work

Contrasted and shallow learning techniques, deep learning has numerous focal points in dissecting pathology images, including clarification of feature definition, power in perceiving complex objects, efficiency through equal calculation, and reasonableness for transfer learning. This paper has tested the EfficientNet model for all variants to classify lung and colon cancer histopathology images. This model aims to scale a CNN model in not just one element but in all three features, namely, depth, width, and resolution, according to available resources in a principled way. This is the first study considering lung and colon image classification and the pretrained EfficientNet model. All variants with different resolutions started at 224 × 224 in the B0 model and increased to 600 × 600 in the B7 model. Each model's last layer is adjusted for better performance, and different dropouts prevent overfitting. The experiment was performed on the LC25000 dataset, having lung and colon images of five classes. The classification accuracy for training the models B0, B1, B2, B3, B4, B5, B6, and B7 are 95.87, 96.26, 97.24, 95.63, 96.83, 94.31, 93.76, and 95.59%. Our model performed well in terms of training time and computational power. We run our model in the minimum time on a personal computer utilizing Google Colab using GPU without accessing a physical GPU attached to the computer. We plan to extend the work by experimenting with more images of different sizes. We also intend to increase the number of classes based on the availability of the data, which may lead to an increase in the accuracy of the model.

## Figures and Tables

**Table 1:** Different image resolutions used for each model.

Base model	Resolution
EfficientNetB0	224 × 224
EfficientNetB1	240 × 240
EfficientNetB2	260 × 260
EfficientNetB3	300 × 300
EfficientNetB4	380 × 380
EfficientNetB5	456 × 456
EfficientNetB6	512 × 512
EfficientNetB7	600 × 600

**Table 2:** Top5 and Top1 accuracy of models trained on the ImageNet dataset.

Model variants	Parameters (m)	Top1 accuracy %	Top5 accuracy %
EfficientNetB0	5.3	76.3	92.3
EfficientNetB1	7.8	78.8	94.4
EfficientNetB2	9.2	79.8	94.9
EfficientNetB3	12	81.1	95.5
EfficientNetB4	19	82.6	96.3
EfficientNetB5	30	83.3	96.7
EfficientNetB6	43	84.0	96.9
EfficientNetB7	66	84.4	97.1

**Table 3:** The model evaluation results in terms of classification loss and accuracy.

Efficient net variants	Training		Validation		Testing		Model training time
	Accuracy	Loss	Accuracy	Loss	Accuracy	Loss	
B0	0.95	0.37	0.93	0.37	0.96	0.34	09 minutes
B1	0.96	0.11	0.96	0.06	0.96	0.11	08 minutes
B2	0.97	0.07	0.07	0.97	0.97	0.07	07 minutes
B3	0.95	0.18	0.95	0.08	0.95	0.18	3 hours 17 minutes
B4	0.96	0.11	0.14	0.94	0.11	0.96	2 hours 33 minutes
B5	0.94	0.33	0.93	0.20	0.95	0.28	36 minutes
B6	0.93	0.22	0.96	0.21	0.94	0.47	2 hours 45 minutes
B7	0.95	3.05	0.92	2.79	0.95	2.75	54 minutes

**Table 4:** Comparison with state-of-the-artwork.

Architectures	Parameters	Input size	Classification	Accuracy result (%)
EfficientNetB4 [29]	17 million	Not specified	Binary and multiclass for COVID-19 diagnosis	96
DCNN [3]	60 million	256 × 256	Three classes of lung cancer subtypes	71.1
Residual neural network [8]	0.27 million	50 × 50	Lung cancer type from CT scan images	85.71
Inception-v3 [13]	23 million	512 × 512	Gastric and colonic from histopathological	96
SC-CNN [30]	Not specified	27 × 27	Nuclei in colon cancer histology images	68
EfficientNetB2 (our approach)	9.2 million	260 × 260	For histopathology images of lung and colon cancer (five classes)	97.24

## Data Availability

Data are available upon reasonable request to the corresponding author.
